# Feasibility of diffusion-weighted magnetic resonance imaging in patients with juvenile idiopathic arthritis on 1.0-T open-bore MRI

**DOI:** 10.1007/s00256-015-2208-3

**Published:** 2015-07-24

**Authors:** Anouk M. Barendregt, Charlotte M. Nusman, Robert Hemke, Cristina Lavini, Dimitri Amiras, Taco W. Kuijpers, Mario Maas

**Affiliations:** Department of Radiology, Academic Medical Center, University of Amsterdam, Meibergdreef 9, 1105 AZ Amsterdam, The Netherlands; Department of Pediatric Hematology, Immunology, Rheumatology and Infectious Disease, Emma Children’s Hospital AMC, University of Amsterdam, Meibergdreef 9, 1105 AZ Amsterdam, The Netherlands; Radiology Department, St. Mary’s Hospital, Imperial College Healthcare NHS Trust, Praed Street, Paddington, London W2 1NY UK

**Keywords:** Diffusion-weighted imaging, Juvenile idiopathic arthritis, Magnetic resonance imaging, Arthritis, Synovial membrane, Knee joint

## Abstract

**Objective:**

To evaluate the feasibility of non-invasive diffusion-weighted imaging (DWI) of the knee of children with juvenile idiopathic arthritis (JIA) and, further, to analyze the apparent diffusion coefficient (ADC) levels to distinguish synovium from effusion.

**Materials and methods:**

Standard magnetic resonance imaging of the knee including post-contrast imaging was obtained in eight patients (mean age, 12 years 8 months, five females) using an open-bore magnetic resonance imaging system (1.0 T). In addition, axially acquired echo-planar DWI datasets (*b*-values 0, 50, and 600) were prospectively obtained and the diffusion images were post-processed into ADC_50–600_ maps. Two independent observers selected a region of interest (ROI) for both synovium and effusion using aligned post-contrast images as landmarks. Mann–Whitney *U* test was performed to compare ADC synovium and ADC effusion.

**Results:**

DWI was successfully obtained in all patients. When data of both observers was combined, ADC synovium was lower than ADC effusion in the ROI in seven out of eight patients (median, 1.92 × 10^−3^ mm^2^/s vs. 2.40 × 10^−3^ mm^2^/s, *p* = 0.006, respectively). Similar results were obtained when the two observers were analyzed separately (observer 1: *p* = 0.006, observer 2: *p* = 0.04).

**Conclusions:**

In this pilot study, on a patient-friendly 1.0-T open-bore MRI, we demonstrated that DWI may potentially be a feasible non-invasive imaging technique in children with JIA. We could differentiate synovium from effusion in seven out of eight patients based on the ADC of synovium and effusion. However, to select synovium and effusion on DWI, post-contrast images were still a necessity.

## Introduction

Juvenile idiopathic arthritis (JIA) is a chronic disease of childhood characterized by joint inflammation [1, 2]. It is the most common rheumatic condition in children with a prevalence rate of 16 to 150 per 100,000 children in Western populations [1]. Common symptoms of JIA are swollen joints, stiffness, pain, and loss of function. Long-lasting disease can lead to joint deformity, growth disturbance, and disability [2].

Imaging plays an important role in the evaluation and follow-up of disease in JIA. Ultrasonography (US) and magnetic resonance imaging (MRI) are both able to depict early signs of disease activity [3, 4] such as synovial hypertrophy and joint effusion. Both imaging modalities can also detect osseous and cartilage lesions [3, 4]. US examination is non-invasive, non-irradiating, and, in comparison to MRI, fast and inexpensive. Disadvantages of US are the inability to image bone marrow edema and non-superficial lesions. Also, there is considerable inter-reader variability and no validated scoring system for pediatric arthritis [4, 5]. Therefore, MRI is considered to be the superior imaging modality. MRI enables detailed evaluation of the affected joint as a whole [3, 4, 6–8] and inter-reader variability is low.

Nonetheless, on 1.0-T MRI, both synovium and joint effusion are hypointense on T1-weighted images and hyperintense on T2-weighted images making differentiation on unenhanced sequences difficult [9]. Contrast-enhanced (CE) MRI can distinguish synovium from effusion and therefore using an intravenous contrast agent is indispensable [10]. The use of contrast in pediatric patients has many drawbacks, mainly because of the invasiveness of intravenous puncture. In addition, administration of contrast is expensive and time consuming. It may seldom cause systemic or local allergic reactions, and rarely acute renal failure or long-term renal fibrosis [11, 12].

These disadvantages of CE MRI indicate the desire for a non-invasive MRI technique. Diffusion-weighted imaging (DWI) is non-invasive and may be suitable to substitute CE MRI. The technique has recently been found useful in musculoskeletal imaging [13–19]. DWI MRI signal is dependent on the diffusion of water molecules. Thus, the signal is amongst others a reflection of the cellularity of the tissue being imaged. In effusion, water molecules move more freely than water molecules within synovium. Thus, we hypothesized that DWI would distinguish synovial tissue from effusion.

The objective of our pilot study is to evaluate DWI in children with JIA and secondly to analyze whether this technique is able to distinguish synovium from effusion based on mean apparent diffusion coefficient levels.

## Materials and methods

### Patients

Consecutive patients included in this study were recruited from the outpatient clinics of two tertiary pediatric rheumatology centers (Academic Medical Center and Reade, both Amsterdam, the Netherlands) and one non-academic pediatric rheumatology center (Sint Lucas Andreas Hospital, Amsterdam, the Netherlands). Recruitment started in February 2013 and the last patient of this pilot study was included in December 2013. The knee is the most commonly affected joint in JIA [20], therefore JIA patients with knee involvement were studied. Patients were eligible for this study if (1) clinically active disease]) was present (defined as a red, warm or swollen knee by the pediatric rheumatologist), and (2) JIA was diagnosed according to the International League of Associations for Rheumatology (ILAR) criteria [21]. All experiments had been conducted after the approval of the institutional medical ethical review board, as well as after obtaining written informed consent. Patients with the following characteristics were not included in the study: age below 8 years or above 18 years, presence of contraindications for MRI scanning (e.g., claustrophobia, need for sedation), recent trauma to the knee, comorbidity concerning the knee joint, or a history of intra-articular corticosteroid injection within the last 6 months. Absence of synovial enhancement and/or effusion on MRI was another exclusion criterion.

### MRI

All patients underwent MRI of the knee with a dedicated knee coil on a patient-friendly 1.0-T open-bore MRI (Panorama HFO, Philips Medical Systems, Best, The Netherlands; slew rate 120 T per meter per second, and gradient amplitude of 28 milliTesla per meter). Patients were in supine position with the knee situated centrally in the MRI. Open-bore MRI was used to increase patient comfort [22]. In addition to standard sequences, an axial DWI sequence was acquired consisting of a T2-weighted single-shot spin-echo echo-planar imaging sequence with b-values 0, 50, and 600. Field of view was from above or at the patella to the proximal tibial epiphysis, the exact borders depending on the size of the knee of the infant. The DWI sequence increased total scanning time with 7 min and 30 s. The DWI sequence was acquired prior to contrast administration: intravenous gadolinium injection (gadobutrol, Gadovist, Bayer Schering Pharma) of 0.1 millimol per kilogram of body weight was administered, and <5 min after contrast administration, contrast-enhanced images were acquired. Parameters of all MRI sequences are listed in Table 1.

**Table 1:** Standard JIA protocol and DWI sequence parameters

	IV Gd	Repetition time (ms)	Echo time (ms)	Field of view	Voxel size (mm)	Slice thickness (mm)	Recon. matrix	TSE factor	NSA	Slice gap	rBW p.p.
Sagittal T2 SPIR	–	2800–4500	50	150 × 150 × 92	0.5 × 0.6	4	445	14	3	0.4	258
Coronal T2 SPIR	–	2800–4500	60	150 × 150 × 92	0.5 × 0.6	4	480	13	3	0.4	240
Axial T2 SPIR	–	2800–4500	50	150 × 150 × 104.8	0.5 × 0.55	4	480	15	3	0.8	188
Axial T2 DWI	–	6282–8164	98–114	180 × 180 × 63	1 × 1	3.5	256	–	8	0	766
Sagittal T1 TSE	–	450–650	10	150 × 150 × 92	0.45 × 0.63	4	480	6	3	0.4	179
Sagittal T1 TSE	+	450–650	10	150 × 150 × 92	0.45 × 0.63	4	480	6	3	0.4	179
Axial T1 SPIR	+	400–750	10	150 × 150 × 104.8	0.55 × 0.77	4	480	6	2	0.8	169

*IV Gd* intravenous gadolinium injection, *ms* millisecond, *mm* millimeter. *Recon matrix* reconstruction matrix, *NSA* number of signal averages, *SPIR* spectral presaturation inversion recovery, *TSE* turbo spin echo, *rBW p.p*. receiver bandwidth per pixel

### Image processing and analysis

**Fig. 1:**
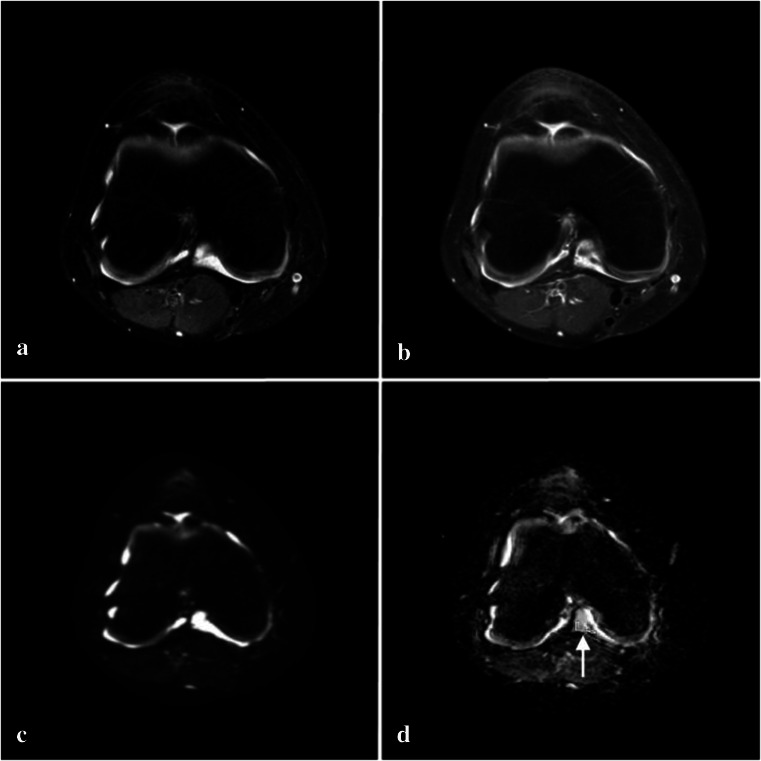
MRI of the knee of an 11-year-old female JIA patient. ROI selection of synovium on ADC_50–600_ map (*arrow points* *to ROI*). **a** Axial T2-weighted SPIR. **b** Post-contrast axial T1-weighted SPIR. **c** Axial diffusion-weighted image. **d** Axial ADC_50–600_ map

**Fig. 2:**
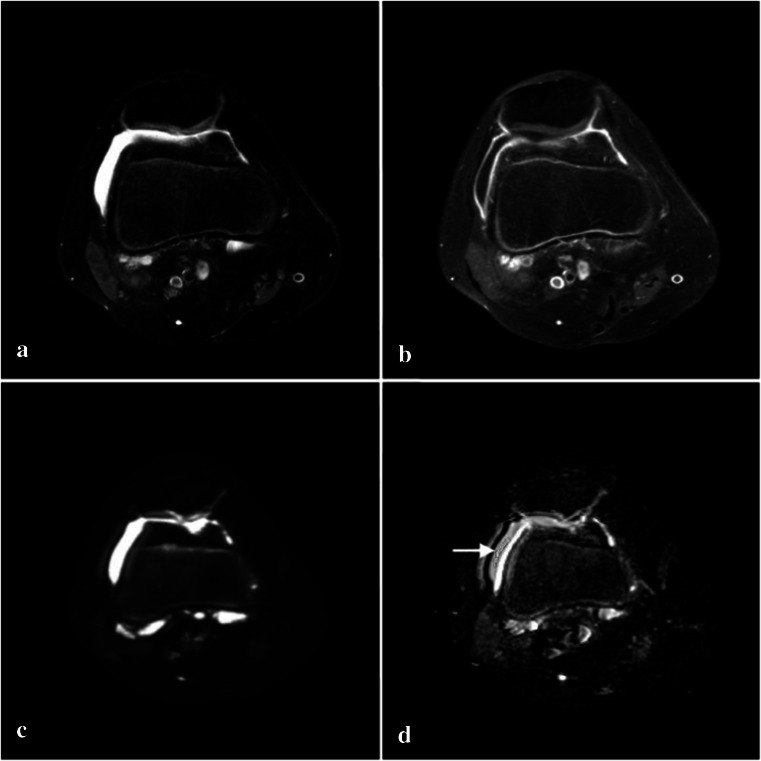
MRI of the knee of an 11-year-old female JIA patient. ROI selection of effusion on ADC_50–600_ map (*arrow points* *to ROI*). **a** Axial T2-weighted SPIR. **b** Post-contrast axial T1-weighted SPIR. **c** Axial diffusion-weighted image. **d** Axial ADC_50–600_ map

All diffusion-weighted images were post-processed into ADC_50–600_ maps on the MRI console computer. The ADC_50–600_ map was chosen to prevent signal from vascular flow to influence the ADC values [23, 24]. For further analysis, in-house-developed software using MATLAB version R2011b (The MathWorks Inc., Natick, MA, USA) was used to assess diffusion. On the ADC maps, a region of interest (ROI) was drawn in a region where synovium was present as demonstrated by CE MRI (Fig. 1), the second ROI was drawn in an area with effusion (Fig. 2). Two independent readers selected ROI (C.M.N., 3 years of experience in musculoskeletal imaging and R.H., 6 years of experience in musculoskeletal imaging). ROI selection on the ADC_50–600_ maps was performed while aligning the post-contrast images to the diffusion-weighted images to ensure correct selection of synovium or effusion as displayed in Figs. 1 and 2. For final confirmation, the ADC maps with ROIs and post-contrast images were discussed under supervision of M.M. (19 years of experience in musculoskeletal imaging); all ROIs were approved. Lastly, mean ADC and standard deviation (SD) of the ROIs were extracted by MATLAB.

### Statistical analysis

Descriptive statistics (medians, range, percentages) and statistical analysis were performed using IBM SPSS Statistics version 19.0 (IBM Corp., Armonk, NY, USA). ADC values of the two observers were averaged ($\frac{ADC\kern0.24em  observer\;1+ADC\; observer\;2}{2}$) to make both a combined analysis (average ADC) and a separate analysis per observer possible. The data had a non-normal distribution, therefore the Mann–Whitney *U* test was performed to assess whether the distribution of ADC synovium was different from ADC effusion. A *p* value of <0.05 was considered statistically significant.

## Results

### Patients

In this feasibility study, 18 patients from different JIA categories met the eligibility criteria. Exclusion because of comorbidity concerning the knee joint (*n* = 2), absence of synovial enhancement on MRI (*n* = 7) and absence of effusion on MRI (*n* = 1) resulted in a total number of eight patients. Among those eight patients were three males and five females, with a mean age of 12 years and 8 months (range, 8.8 – 15.8 years). Patient characteristics are summarized in Table 2.

**Table 2:** Patient characteristics

Patient	Gender	JIA category	Age at inclusion (years, months)
1	Female	Psoriatic arthritis	14.4
2	Female	Oligoarthritis	15.8
3	Female	Oligoarthritis	11.5
4	Female	Polyarthritis (RF+)	13.7
5	Male	Oligoarthritis	11.7
6	Male	Polyarthritis (RF-)	8.8
7	Male	Polyarthritis (RF+)	13.2
8	Female	Polyarthritis (RF-)	14.4

JIA categories as defined by ILAR

*RF+* rheumatoid factor positive, *RF-* rheumatoid factor negative

### Feasibility of the DWI sequence

Diffusion-weighted images were successfully obtained in all patients that were included in the study. Minor motion and fold-over artifacts were observed, but interpretation of the diffusion-weighted images was not hampered and no MRI dataset was excluded.

## ADC results

**Fig. 3:**
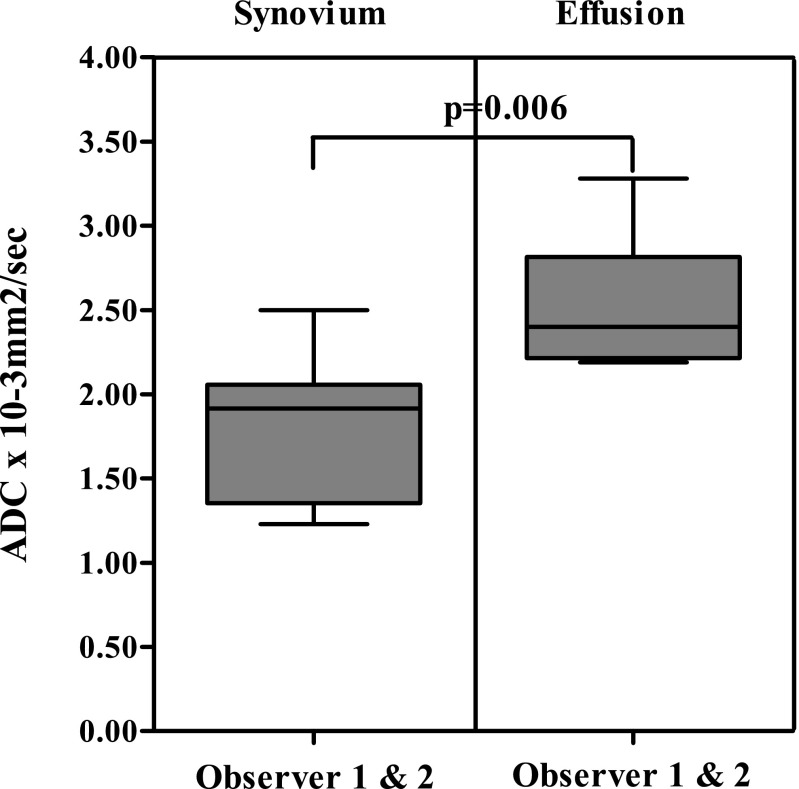
Boxplot of total ADC synovium and total ADC effusion. *p* value of the Mann–Whitney *U* test is shown

**Fig. 4:**
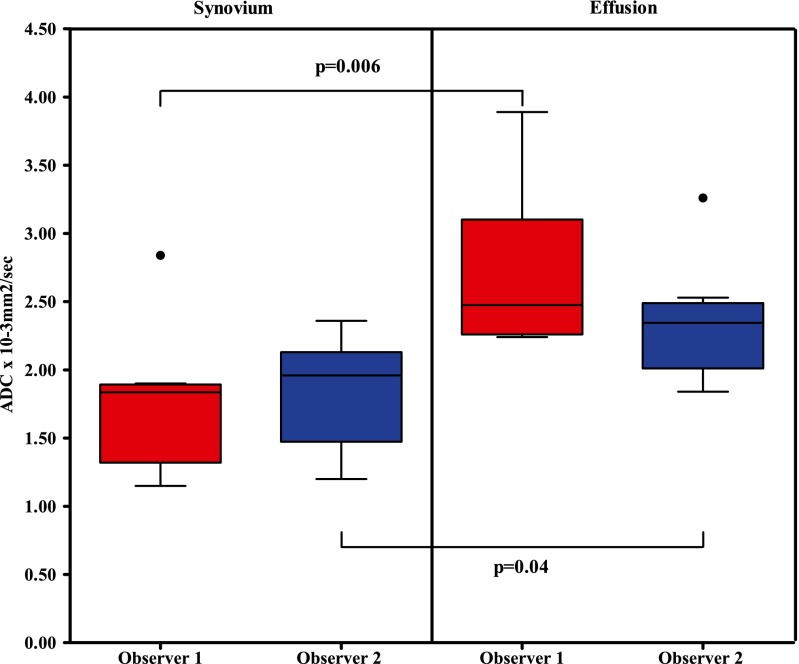
Boxplot of ADC synovium and ADC effusion, separated per observer. *p* values of the Mann–Whitney *U* test are also shown for both observers. *Circle* (●) = outlier, ADC higher than 1.5× interquartile range (IQR) above the third quartile (Q_3_), in formula: ADC value > Q_3_+ 1.5 × IQR

**Fig. 5:**
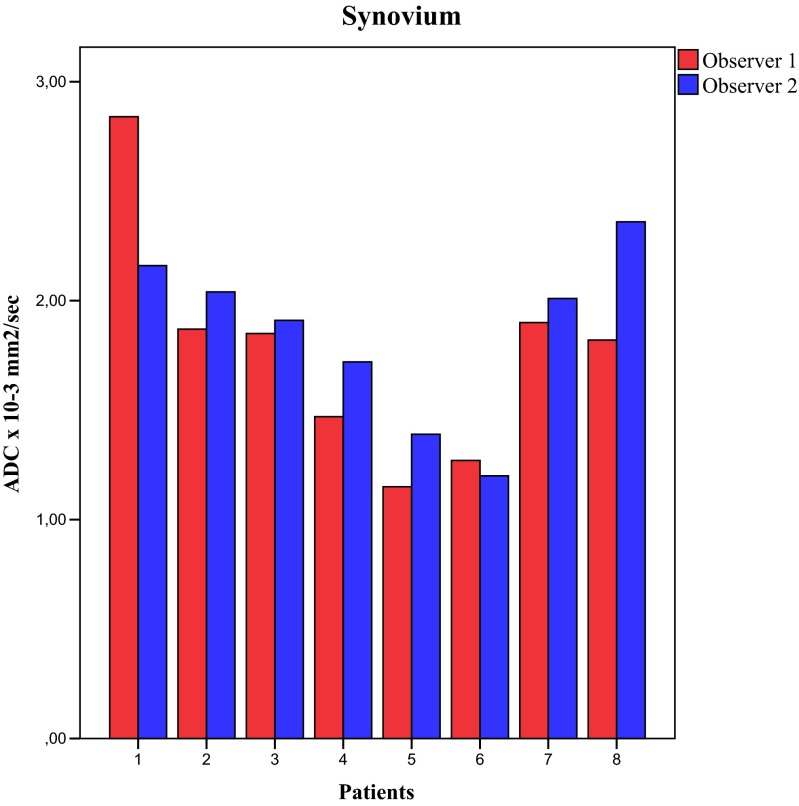
Histogram of paired ADC values of synovium per observer

**Fig. 6:**
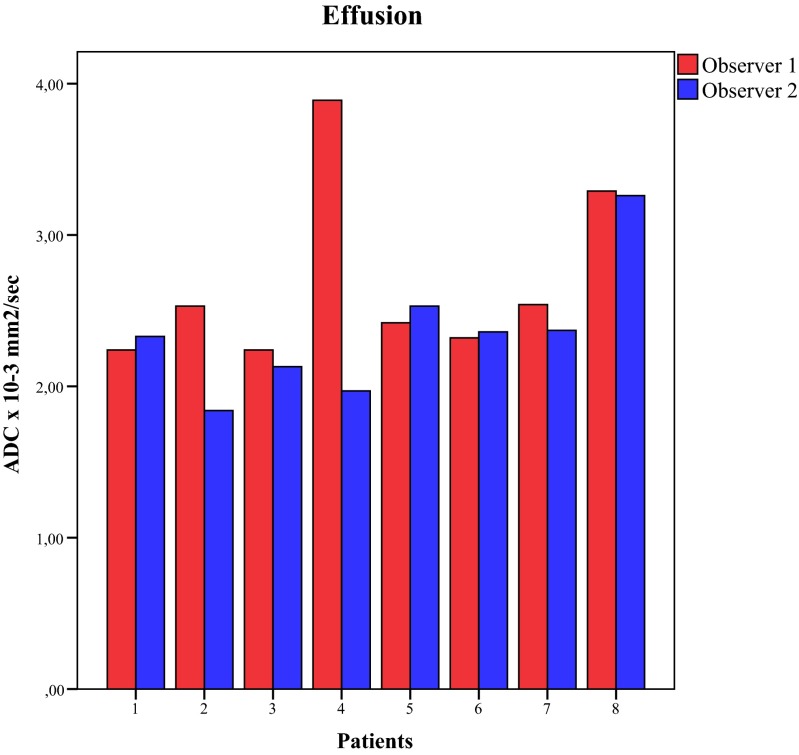
Histogram of paired ADC values of effusion per observer

The ADC values of all patients are shown in Table 3. The analysis of the combined ADC values showed that median ADC values of synovium were significantly lower compared to the median ADC values of effusion (1.92 × 10^−3^ mm^2^/s vs. 2.40 × 10^−3^ mm^2^/s, *p* = 0.006, respectively). A boxplot of these data is shown in Fig. 3. In the per-observer analysis (Fig. 4), observer 1 had a median synovial ADC of 1.84 × 10^−3^ mm^2^/s and a median effusion ADC of 2.48 × 10^−3^ mm^2^/s (*p* = 0.006). For observer 2, these values were 1.96 × 10^−3^ mm^2^/s and 2.35 × 10^−3^ mm^2^/s, respectively (*p* = 0.04). In seven out of eight patients, ADC synovium was lower than ADC effusion. This was valid for both the averaged ADC (both observers together) and the separate analysis of the two observers (Figs. 5 and 6).

**Table 3:** Mean ADC of the ROI synovium and mean ADC of the ROI effusion of all patients

Patient	ADC synovium (×10^−3^ mm^2^/s)			ADC effusion (×10^−3^ mm^2^/s)		
	Observer 1	Observer 2	Average	Observer 1	Observer 2	Average
1	2.84	2.16	2.50	2.24	2.33	2.29
2	1.87	2.04	1.96	2.53	1.84	2.19
3	1.85	1.91	1.88	2.24	2.13	2.19
4	1.47	1.72	1.60	3.89	1.97	2.93
5	1.15	1.39	1.27	2.42	2.53	2.47
6	1.27	1.20	1.23	2.32	2.36	2.34
7	1.90	2.01	1.95	2.54	2.37	2.46
8	1.82	2.36	2.09	3.29	3.26	3.28

## Discussion

In this pilot study, we evaluated the feasibility of DWI in JIA patients with active disease at the time of imaging. Firstly, our results demonstrate that DWI is a feasible technique in this pediatric population. For the majority of patients, we were able to distinguish synovium from effusion, since ADC synovium was lower than ADC effusion in seven out of eight patients.

Recently, the use of DWI in musculoskeletal imaging has been explored by several authors [14–19]. The results of these studies show that DWI can be of added value for imaging a diverse variety of musculoskeletal disorders. Only one of these studies evaluated DWI in JIA patients: Neubauer and colleagues described the ADC of osseous lesions, soft tissue edema, joint effusion, and synovitis in a population of patients with non-tumorous musculoskeletal lesions [19]. However, the results of this study cannot be compared directly to our results because our study included only JIA patients, while the Neubauer study included a heterogeneous group of 52 patients with various musculoskeletal lesions such as osteomyelitis, tuberculous coxarthritis, and traumatic lesions as well as JIA. In addition, it is difficult to directly compare the ADC values from our study to the diffusion coefficients in the recent study of Neubauer et al., since scanning parameters, magnetic field strength, and MR vendor were different. Also, different B values were used (ADC_0–1000_ map) [19], whereas we deliberately used the ADC_50–600_ map to exclude bias from vascular flow in the MRI signal. Other studies evaluated the use of DWI for imaging synovial inflammation in rheumatoid arthritis (RA) [25, 26]. Jeromel et al. [25] studied RA patients with synovitis in the cranio-cervical region by selecting a region of interest around the odontoid process. They included 27 patients with early RA and performed a baseline MRI and a follow-up MRI at 6 months. Dynamic CE MRI and DWI were performed next to standard qualitative MRI, and the results indicate that DWI is feasible in this anatomically complex region [25]. Another feasibility study of 25 patients with RA in the hand and wrist found DWI to detect synovitis more accurately than T2-weighted MRI with short-tau inversion recovery, however DWI was less accurate than CE MRI. In this study, DWI was used qualitatively (‘high signal intensity at high b-value’ was scored as synovitis), which complicates a comparison with our quantitative results [26].

There are several limitations in our study. First of all, distinguishing synovium from effusion was not possible on the ADC maps alone, hence the ADC maps were cross-referenced with post-contrast images to ensure correct selection of synovium and effusion. This is probably due to a low signal-to-noise ratio (SNR) and scanning at a higher field strength is expected to increase SNR and reduce movement artifacts as a result of decreasing scan duration. Increased SNR and (minor to absent) motion artifacts will make contrast between synovium and effusion stronger, thereby making selection of synovium and effusion easier and more reliable. These studies have been planned. Secondly, the number of patients for DWI analysis (*n* = 8) was small. A further limitation in our study is that in some patients the ADC of synovium was higher as compared to the ADC of joint effusion (Table 3, patients 1 and 2). A possible explanation for these unexpected results could be that for patient 1 the ROI included an arteriole, venule, or even effusion, or the ROI of synovium could have been too large. Regarding the ROI of the effusion in patient 2, the joint fluid might have had a high protein concentration, or a small rim of non-fluid tissue could have been incorporated, which could have decreased the ADC. Scanning with increased resolution is expected to reduce the effect of this partial voluming. A final limitation to mention is that despite a fair degree of concordance in ADC values, the ADC scores of the two observers were not similar for some patients. Future studies with more patients should help to verify whether this was due to a learning curve in the interpretation or additional methodological issues of the analysis.

In conclusion, diffusion-weighted imaging might have the potential of replacing CE MRI for the assessment of synovial inflammation, leading to increased patient comfort, safety and compliance, and reduced costs in the imaging of children with JIA. However, further evaluation with higher field strengths and increased patient numbers is needed to perfect the technique since post-contrast images were a necessity to correctly select synovium and effusion on DWI. The results of our study demonstrate the potential feasibility and applicability of DWI in this pediatric population and show the ability of DWI to quantitatively distinguish synovial tissue from effusion using ADC values.
